# Anisokorie mal anders

**DOI:** 10.1007/s00347-020-01153-y

**Published:** 2020-06-25

**Authors:** Jan Klonner, Daniel Salchow

**Affiliations:** 1 Universitäts-Augenklinik, Charité – Universitätsmedizin Berlin, Augustenburger Platz 1, 13353 Berlin, Deutschland; 2 Universitäts-Augenklinik, Leitung der Sektion Kinderaugenheilkunde, Strabologie/Orthoptik, Neuroophthalmologie, Charité – Universitätsmedizin Berlin, Augustenburger Platz 1, 13353 Berlin, Deutschland

**Keywords:** Anisokorie, Iristransillumination, Infekt der oberen Atemwege, BAIT-Syndrom, Fluorchinolone, Anisocoria, Iris-Transillumination, Upper respiratory tract infection, BAIT-Syndrome, Fluoroquinolone

## Abstract

Eine 53-jährige Patientin beklagte erhöhte Blendempfindlichkeit 3 Wochen nach Einnahme von Moxifloxacin-Tabletten bei Infekt der oberen Atemwege. Es bestand eine Anisokorie, die Pupillenreaktion, sowohl auf Licht als auch auf Naheinstellung, war aufgehoben. In der Untersuchung des vorderen Augenabschnittes fielen beidseits ausgeprägte Iristransilluminationsdefekte (ITD) auf. Wir diagnostizierten ein BAIT-Syndrom (bilaterales akutes Iristransilluminationssyndrom). Dies ist ein seltenes Syndrom, welches mit einer massiven Depigmentierung der Iris sowie einer Atrophie der Irismuskulatur einhergeht. Risikofaktor für die Entstehung eines BAIT-Syndroms scheint die orale Einnahme von Antibiotika, insbesondere Moxifloxacin, im Rahmen eines Infektes der oberen Atemwege zu sein, aber auch spontan auftretende Fälle sind beschrieben. Betroffen sind v. a. Frauen mittleren Alters. Die genaue Ursache des BAIT-Syndroms ist bisher unklar. Diskutiert wird ein möglicher Einfluss der Konzentration des Antibiotikums im Glaskörper. Differenzialdiagnostisch muss bei Iristransilluminationsdefekten insbesondere auch an Albinismus, intraokuläre Entzündungen, Pseudoexfoliationssyndrom und Pigmentdispersionssyndrom gedacht werden. Eine spezifische Therapie des BAIT-Syndroms besteht bisher nicht. Erhöhte Lichtempfindlichkeit und ein Post-BAIT-Glaukom können mögliche Komplikationen sein. Die Kenntnis des seltenen BAIT-Syndroms kann im klinischen Alltag hilfreich bei der differenzialdiagnostischen Einordnung einer Anisokorie sein und ggf. zur Vermeidung unnötiger diagnostischer Schritte beitragen.

## Abstract (en)

A 53-year-old female patient presented with increased light sensitivity 3 weeks after oral intake of moxifloxacin tablets for an upper respiratory tract infection. The symptoms were anisocoria and the pupils did not react to light or accommodation. The examination of the anterior segment of the eye revealed extensive bilateral iris transillumination defects (ITD). We diagnosed a bilateral acute iris transillumination (BAIT) syndrome. The BAIT syndrome is a rare disorder associated with massive depigmentation of the iris and atrophy of the iris musculature. A risk factor for BAIT syndrome seems to be the oral intake of antibiotics, in particular moxifloxacin after an upper respiratory tract infection but cases of spontaneous occurrence have also been described. Middle-aged women are particularly affected. The exact cause of BAIT syndrome is so far unknown but a potential mechanism involves the concentration of the antibiotic in the vitreous body. Differential diagnoses include other causes for ITD, such as albinism, intraocular inflammation, pseudoexfoliation syndrome and pigment dispersion syndrome. To date there is no specific treatment for BAIT syndrome. Possible complications include increased light sensitivity and post-BAIT glaucoma. Knowledge of the rare BAIT syndrome can be useful in the clinical routine for the differential diagnostic classification of an anisocoria and can possibly contribute to avoidance of unnecessary diagnostic steps.

## Anamnese

Eine 53 Jahre alte Patientin stellte sich erstmals im Mai 2018 in unserer Rettungsstelle vor.

Sie berichtete, ihre Kosmetikerin habe am selben Tag eine Größendifferenz der Pupillen festgestellt.

In der Anamnese berichtete die Patientin von einer erhöhten Blendempfindlichkeit in den letzten Wochen. Die sonstige Augenanamnese blieb bis auf eine bekannte Myopie leer. Auch vorangegangene Operationen an den Augen wurden verneint. In der Allgemeinanamnese gab die Patientin eine allergische Bronchitis an. Aufgrund einer Stenose der A. carotis interna links befand sich die Patientin in neurologischer Abklärung. Aus diesem Grund wurde eine orale Therapie mit Acetylsalicylsäure 100 mg täglich prophylaktisch begonnen.

Die Patientin berichtete zudem, etwa 3 Wochen vor der Vorstellung bei uns während eines Urlaubsaufenthaltes in Italien an einer Sinusitis erkrankt zu sein. Diese wurde mit Moxifloxacin 400 mg 1‑mal täglich per os therapiert. Die Patientin habe die Tabletten bei subjektiver Beschwerdebesserung am dritten Tag der Behandlung abgesetzt.

## Klinischer Befund

Der bestkorrigierte Visus war dezimal 1,0 an beiden Augen (Refraktionsfehler rechtes Auge: −2,25 sph −0,5 zyl./75°, linkes Auge: −1,75 sph.). Der Augeninnendruck betrug rechts 21 mm Hg und links 17 mm Hg (Goldmann-Applanationstonometrie). Die Bulbusmotilität war beidseits frei, eine Ptosis fehlte.

Die Bindehaut war beidseits reizfrei, die Hornhaut klar und glatt. Die Vorderkammer war beidseits tief, ohne Zeichen einer aktiven oder abgelaufenen Entzündung. Der Kammerwinkel zeigte sich in der Gonioskopie an beiden Augen mäßig pigmentiert und offen (Grad II–III nach Schaffer).

**Figure 1:**
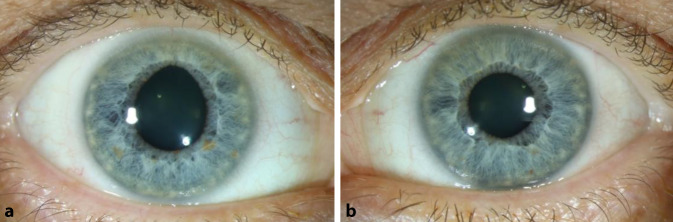

Es zeigte sich eine Anisokorie (rechte Pupille größer als die linke) bei beidseits mittelweiter Pupille. Weder eine direkte noch indirekte Lichtreaktion war zu beobachten, die rechte Pupille war nach 12 Uhr verzogen (Abb. 1).

**Figure 2:**
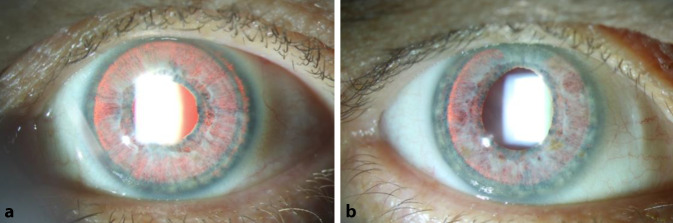

Im retrograden Licht fielen an beiden Augen ausgeprägte Transilluminationsdefekte der Iris auf (Abb. 2). Fundoskopisch zeigte sich beidseits ein regelrechter Makulabefund inklusive unauffälliger Fovea. Die Papille war beidseits eher klein und mit myopem Konus sowie einer Cup-Disc-Ratio von 0,2 ebenfalls unauffällig.

Die Netzhautperipherie wies keine pathologischen Veränderungen auf. In der Pupillometrie konnte an beiden Augen keine messbare Änderung der Pupillengröße im Hellen und Dunklen festgestellt werden.

Zur weiteren pharmakologischen Diagnostik applizierten wir Pilocarpin 0,1 %-Augentropfen in beiden Augen. Es zeigte sich auch hier keine Veränderung der Pupillenweite, sodass eine Pupillotonie sowie eine Adie-Pupille ausgeschlossen werden konnten.

### Was ist Ihre Verdachtsdiagnose?

Bilaterales akutes Iristransilluminationssyndrom (BAIT).

## Auflösung und Diskussion

Die Patientin hat ein bilaterales akutes Iristransilluminationssyndrom (BAIT). Das BAIT-Syndrom wurde erstmals im Jahr 2004 beschrieben [1]. Es finden sich gehäuft Fälle aus der Türkei und Belgien [8]. Vorwiegend sind mittelalte Frauen betroffen (75 % der Fälle sind weiblich) [6], die meist eine vorangegangene Infektion der oberen Atemwege hatten, es sind jedoch auch Fälle nach Harnwegsinfektion und ohne infektiöse Vorgeschichte beschrieben. Die Mehrzahl der Patienten mit BAIT-Syndrom hatten orale, oder parenterale Antibiotika erhalten (in 81 %), zumeist Moxifloxacin (in 66 %) [6]. Extrem selten ist das BAIT-Syndrom nach topischer Anwendung von Moxifloxacin-Augentropfen über eine mittlere Dauer von 10 Tagen beschrieben (2 Fälle) [7]. Das BAIT-Syndrom ist durch eine massive Depigmentierung der Iriden und oft durch einen signifikanten Anstieg des intraokularen Druckes gekennzeichnet [6]. Letzterer fehlte bei unserer Patientin, vielleicht, weil sie sich erst spät nach subjektivem Symptombeginn bei uns vorstellte. Eine trabekuläre Hyperpigmentierung (fehlte bei unserer Patientin), die offenbar mit der Zeit abnimmt, sowie eine pupilläre Atonie mit halbweiter Pupille, die schwach oder gar nicht auf Licht reagiert, sind ebenfalls typisch. Bei unserer Patientin fanden wir auch eine bisher nicht beschriebene fehlende Konstriktion der Pupille auf Naheinstellung. Konjunktivale Hyperämie (100 % der Fälle), Photophobie (88 %), Augenschmerzen (38 %) und Verschwommensehen (27 %) sind weitere Merkmale des BAIT-Syndroms [8]. Des Weiteren wurde von pigmentierten endothelialen Präzipitaten, manchmal in Form einer Krukenberg-Spindel, und initial pigmentierten Zellen in der Vorderkammer berichtet [9].

Transilluminationsdefekte der Iris können auch bei Albinismus auftreten, dieser konnte bei normalem Visus, fehlendem Nystagmus, normaler Funduspigmentierung und Fovea ausgeschlossen werden. Okuläre Infektionen mit Irisatrophie und Depigmentierung, wie beispielsweise eine Fuchs-Uveitis, oder eine Herpes-simplex-assoziierte Uveitis sind bei symmetrischem Befund, fehlendem Reizzustand sowie bei fehlenden Anzeichen einer abgelaufenen intraokularen Entzündung unwahrscheinlich. Beim Pseudoexfoliationssyndrom (PEX-Syndrom) ist die Depigmentierung der Iris meist auf den peripupillären Bereich begrenzt. Die für ein PEX-Syndrom ebenfalls typischen schuppigen Ablagerungen auf der Linsenvorderfläche hatte unsere Patientin nicht. Auch ein Pigmentdispersionssyndrom kommt differenzialdiagnostisch in Betracht, unterscheidet sich aber durch den chronischen und milderen Verlauf sowie durch die konkave Oberfläche der Iris vom BAIT-Syndrom.

Bei einer pathologischen Lichtreaktion der Pupille sollte man neben einer pharmakologischen Mydriasis eine Pupillotonie und ein Horner-Syndrom ausschließen. Bei einer fehlenden Pupillenreaktion aufgrund einer internen Ophthalmoplegie ist zusätzlich die Akkommodation herabgesetzt. Ähnlich wie das BAIT-Syndrom, aber milder, präsentiert sich das BADI-Syndrom (bilaterale akute Depigmentierung der Iris; hier fehlen Transilluminationsdefekte), welches 2006 erstmalig beschrieben wurde [10]. Möglicherweise handelt es sich um verschiedene Ausprägungen derselben Erkrankung.

Neben der Depigmentierung findet sich auch eine Unterfunktion der Irismuskulatur, was sich in der herabgesetzten Pupillenreaktion auf Licht und Akkommodation widerspiegelt. Damit sind nicht nur das Pigmentblatt der Iris, sondern auch Muskelfasern betroffen.

Wodurch das BAIT-Syndrom verursacht wird, konnte bisher nicht vollständig geklärt werden. Ein wichtiger Risikofaktor scheint die systemische Gabe von Antibiotika (insbesondere von Moxifloxacin) bei Infekten der oberen Atemwege zu sein [2–4]. Pharmakologische Untersuchungen legen nahe, dass eine hohe Moxifloxacin-Konzentration im Glaskörper die Entstehung eines BAIT-Syndroms begünstigt. Diese konnte bei Kaninchen nach systemischer, nicht aber nach topischer Gabe nachgewiesen werden [5].

Als Komplikation ist des Weiteren ein Post-BAIT-Glaukom beschrieben, das bleibende Ausfälle der retinalen Nervenfaserschicht hinterlassen und in Einzelfällen eine chirurgische Intervention notwendig machen kann [9].

## Therapie

Eine kausale Therapie nach Diagnose des BAIT-Syndroms ist nicht beschrieben. Prophylaktisch ist ein promptes Absetzen von (Fluorchinolon‑)Antibiotika bei Vorliegen der oben beschriebenen Anzeichen sinnvoll, sofern dies möglich ist. Ob dies den Verlauf mildert, ist nicht bekannt. Ein erhöhter Augeninnendruck sollte adäquat behandelt werden. Bei Photophobie können getönte Brillengläser mit UV-Schutz verordnet werden.

## Fazit für die Praxis

- Nach systemischer Gabe von Antibiotika bei Atemwegsinfekten (insbesondere Fluorchinolonen) kann es zu einer bilateralen akuten Transillumination der Iriden kommen (BAIT-Syndrom).
- Bisher ist die Ursache dieses Phänomens nicht eindeutig geklärt. Komplikationen wie Augendruckanstieg und erhöhte Blendempfindlichkeit sollten symptomatisch behandelt werden.
- Die Kenntnis des BAIT-Syndroms hilft, unnötige Diagnostik bei Anisokorie und reduzierter Pupillenreaktion zu vermeiden und betroffene Patienten angemessen zu beraten.

## References

[1] Bringas Calvo R, Iglesias Cortiñas D (2004). Acute and bilateral uveitis secondary to moxifloxacin. Arch Soc Esp Oftalmol.

[2] Hinkle DM, Dacey MS, Mandelcorn E (2012). Bilateral uveitis associated with fluoroquinolone therapy. Cutan Ocul Toxicol.

[3] Kreps EO, Hondeghem K, Augustinus A (2017). Is oral moxifloxacin associated with bilateral acute iris transillumination?. Acta Ophthalmol.

[4] DeLaney MC (2018). Risks associated with the use of fluoroquinolones. Br J Hosp Med (Lond).

[5] Fukuda M, Shibata N, Osada H, Yamashiro Y, Sasaki H (2011). Vitreous and aqueous penetration of orally and topically administered moxifloxacin. Ophthalmic Res.

[6] Perone JM, Chaussard D, Hayek G (2019). Bilateral acute iris transillumination (BAIT) syndrome: literature review. Clin Ophthalmol.

[7] Kawali A, Mahendradas P, Shetty R (2019). Acute depigmentation of the iris: a retrospective analysis of 22 cases. Can J Ophthalmol.

[8] Tugal-Tutkun I, Onal S, Garip A (2011). Bilateral acute iris transillumination. Arch Ophthalmol.

[9] Den Beste KA, Okeke C (2017). Trabeculotomy ab interno with trabectome as surgical management for systemic fluoroquinolone-induced pigmentary glaucoma: a case report. Medicine (Baltimore).

[10] Tugal-Tutkun I, Urgancioglu M (2006). Bilateral acute depigmentation of the iris. Graefes Arch Clin Exp Ophthalmol.

